# Data on dendrometric parameters, basic wood density, below- and aboveground biomass of tree species from Mangrove, Miombo, Mopane, and Mecrusse woodlands

**DOI:** 10.1016/j.dib.2020.105154

**Published:** 2020-01-19

**Authors:** Tarquinio Mateus Magalhães, Victoria Norberto Cossa, Rosta Simão Mate

**Affiliations:** Departamento de Engenharia Florestal, Universidade Eduardo Mondlane, Campus Universitário, Edifício no. 1, 257 Maputo, Moçambique

**Keywords:** Mozambique's forest ecosystems, Tree and forest biomass, Miombo, Mopane, Mecrusse, Mangrove

## Abstract

Mozambique is composed by the following forest types: Miombo, Mopane, Mecrusse, and Mangrove. Data on basic wood density at different height levels, tree component dry-mass, and other dendrometric parameters (root collar diameter, diameter at breast height, crown height, crown diameter, live crown length, and stem volume) for eight species typical of Miombo (*Afzelia quanzensis* Welw., *Millettia stuhlmannii* Taub., *Pterocarpus angolensis* DC., *Brachystegia spiciformis* Benth., and *Julbernardia globiflora* (Benth.) Troupin), Mopane (*Colophospermum mopane* Kirk ex J. Léonard), Mecrusse (*Androstachys johnsonii* Prain), and Mangrove (*Avicennia marina* (Forssk.)) forests collected from five provinces (Maputo, Gaza, Inhambane, Sofala, and Manica) of Mozambique are presented in this article. Biomass data of Miombo, Mecrusse, and Mopane woodlands were collected destructively, whereas those of Mangrove forests were collected using non-destructive methods.

**Table:** Specifications Table

Subject area	*Agricultural and biological sciences*
More specific subject area	*Forestry. Forest management and modelling.*
Type of data	*Table*
How data were acquired	*Biomass data and related dendrometric variables of Miombo, Mecrusse, and Mopane woodlands were collected destructively, whereas those of Mangrove forests were collected using non-destructive sampling methods.*
Data format	*Raw*
Parameters for data collection	*The following dendrometric variables were measured from each tree of each species, forest type and region: diameter at breast height, root collar diameter, total tree height, stem height, crown height, crown diameter, live crown length, belowground biomass, stem biomass, branch biomass, foliage biomass, crown biomass, basic wood density at different stem height levels, basic wood density of the thickest branch, and stem volume.*
Description of data collection	*Two-phase sampling design was used. In the first phase, trees were measured for different dendrometric variables (root collar diameter, diameter at breast height, tree height, live crown length, and crown radius) within sampling plots. In the second phase, 2 to 6 trees representing all size classes found within each plot were selected for destructive or non-destructive biomass sampling and re-measurement of the phase-1 variables. Samples were collected and taken to the laboratory for dry mass determination and subsequent computation of tree component biomass and basic wood density.*
Data source location	*Mozambique (18°15′00″S, 35°00′00″E): Maputo (25°58′00″S, 32°34′60″E), Inhambane (23°51′53″S, 35°22′59″E), Gaza (23°44′60″S, 32°44′60″E), Sofala (21°08′35″S, 34°16′17″E), Manica (19°30′00″S, 33°15′00″E) provinces.*
Data accessibility	*Mendeley Data*: https://data.mendeley.com/datasets/f8rx3dhjhx/draft?a=1c23bbed-d1b1-4fc3-8d08-b412f91ce965

**Table:** 

**Value of the Data** • Here are presented various dendrometric parameters for all indigenous forest types of Mozambique: Miombo, Mopane, Mecrusse, and Mangroves. • Tree biomasses presented here can be used to fit site- and species-specific below- and aboveground biomass models and biomass expansion and conversion factors which are very important to estimate carbon pools in forests. • The biomass data presented here is a crucial ecological variable for understanding the evolution and potential future changes of the climate system, to assess nutrient cycling and fluxes and energy wood potentials, etc. • The basic density is a useful measure of quality of the wood and can be used to infer about the decomposition rate of the wood and wood debris of each species. • The data can be used to develop models to predict wood density variation using species and position on the stem as independent variables. These models and the wood density itself can be used to estimate carbon stored in woody stems of trees.

## 1 Data description

Reported here are the original data related to the following articles: (1) “Least squares-based biomass conversion and expansion factors best estimate biomass than ratio-based ones: Statistical evidences based on tropical timber species” [1], and (2) “Carbon storage in secondary mangroves along the West coastline of Maputo city, Mozambique” [2]; including other unpublished material. The data are presented as a.xlsx file, composed by two sheets named (1) Data, and (2) Legend. The sheet named Data contains tree component biomasses and other dendrometric parameters of all forest types of Mozambique (Miombo, Mopane, Mecrusse, and Mangrove). The sheet named Legend contains the full names of the abbreviations and dendrometric symbols used in the sheet named Data.

## 2 Experimental design, material and methods

### 2.1 Sampling design and measurement of dendrometric variables

Two-phase sampling design was used for data collection. In the first phase, sampling plots (72, 77, 30, and 10 for Mangrove, Miombo, Mecrusse, and Mopane, respectively) were randomly distributed in the study area. The size of the sampling plots differed in each forest type: 100 m × 20 m, 20 m × 20 m, 20 m radius plots were used for Miombo, Mangrove, and Mecrusse and Mopane, respectively. Root collar diameter (RCD), diameter at breast height (DBH), total tree height, live crown length (LCL), crown radius, and crown diameter (CD) were measured for all target trees within the sampling plots. Target trees were defined as those with RCD ≥10 mm for Mangrove forests, and DBH ≥50 mm for other forest types. RCD and DBH were measured with the aid of caliper or a caliper rule and the total tree heights were measured using a telescopic measuring pole, a ruler or Vertex IV hypsometer. A right-angle prism densiometer and a tape were used to measure crown radius. Crown radius was measured from the centre of the trunk to the perimeter of the crown, in four cardinal directions (North, South, East and West). CD was calculated as double of the geometric mean crown radius. In the second phase, 1 to 6 trees representing all RCD and or DBH classes and all species found within each plot were selected for destructive or non-destructive biomass measurement, according to the case.

### 2.2 Destructive biomass sampling

Destructive sampling was carried out for tree species from Miombo (*Afzelia quanzensis* Welw., *Millettia stuhlmannii* Taub., *Pterocarpus angolensis* DC., *Brachystegia spiciformis* Benth., and *Julbernardia globiflora* (Benth.) Troupin), Mopane (*Colophospermum mopane* Kirk ex J. Léonard) and Mecrusse (*Androstachys johnsonii* Prain) forests.

A total of 235 trees with DBH ≥50 mm were destructively harvested. Of the harvested tree species 121, 34, 24, 24, 19, 17, and 15 were from *A. johnsonii* (DBH range: 50.0–320.0 mm)*, B. spiciformis* (DBH range: 51.5–340.5 mm)*, J. globiflora* (DBH range: 50.0–340.5 mm)*, A. quanzensis* (DBH range: 135.0–611.0 mm)*, P. angolensis* (DBH range: 140.0–465.0 mm)*, C. mopane* (DBH range: 50.0–1092.0 mm), and *M. stuhlmannii* (DBH range: 210.0–522.0 mm), respectively.

Trees were felled considering a predefined stump height of 20 cm. The shoot system was partitioned into following biomass compartments: stem, branches, foliage, and crown (branches + foliage), except for *B. spiciformis* and *J. globiflora*, for which the crown was not divided into branches and foliage. The stem was defined as the length from the top of the stump to the height corresponding to 2.5 cm diameter, although for Miombo species, in which stem length is limited by branching and bifurcation (forking), the natural top of 2.5 cm could not be easily defined.

The stem was divided into 5 segments with length proportional to the stem length, i.e. each segment was one fifth of the stem length. The diameter of each segment was measured at midpoint of it (i.e. at height levels of 10, 30, 50, 70, and 90% of stem). After fresh-weighting the segments, a disc sample was removed at the top of each segment (i.e. at height levels of 20, 40, 60, 80, and 100% of stem), at the base of the first segment (at height level of 0% of stem), and at breast height (1.3 m from the ground level). The disc samples were fresh-weighted in the field, packed in plastic bags and taken to the laboratory. In the laboratory the discs were oven-dried at 105 °C until constant mass and then dry-weighted. The dry mass of each segment was calculated using the ratio between dry- and fresh mass of the discs, multiplied by the fresh mass of the respective segment. Stem biomass was obtained as the sum of the dry masses of the constituent segments. Stem volume was computed using Hohenadl's formula [3].

The dry mass of foliage and branches of all species, except *B. spiciformis and J. globiflora* were obtained as follows: first all the leaves were removed from the branches. Each leafless primary branch, including its secondary, high-order branches, and twigs, was fresh-weighted in the field and a representative sample taken to the laboratory. The sample was made up of a disc removed from the primary branch, samples of secondary and higher-order branches and twigs. The foliage was fresh-weighted in the field and a sample of ≈5% of the fresh mass taken to the laboratory. Dry mass of each primary branch and that of the foliage was obtained similarly to that of each stem segment.

For logistical reasons and to ensure the largest sample size, the foliage of *B. spiciformis* and *J. globiflora* was not separated from the branches. The crown was fresh-weighted and a sample comprising the branches (fine and coarse), twigs, leaves, flowers and fruits was collected, fresh-weighted in the field, and oven-dried in the laboratory. The dry mass of the crown (crown biomass) was obtained similarly to that of each stem segment.

Belowground biomass was only determined for *A. johnsonii* trees. The root system was excavated to total depth, removed and divided into 3 sub-components: fine lateral roots, coarse lateral roots, and taproot. Refer to Magalhães [4] and Magalhães and Seifert [3] for more details on root biomass sampling. Lateral roots with diameters at insertion point on the taproot <5 cm were considered as fine roots and those with diameters ≥5 cm were considered as coarse roots. Fine lateral roots, and coarse lateral roots were sampled and its dry masses determined similarly as foliage and primary branches. For the taproot the sample was composed by two discs: one removed immediately below the ground level and another one removed in the middle of the taproot. The dry-mass was obtained by multiplying the ratio of oven-dry- to fresh mass of the sample by the fresh mass of the taproot.

### 2.3 Non-destructive biomass sampling

Non-destructive biomass sampling was carried out for *Avicennia marina* (Forssk.), from Mangrove forests. A total of 301 saplings and trees (RCD range: 10.0–160.0 mm) were non-destructively sampled.

The standing tree stem was divided into 5 segments with length proportional to the stem height (i.e. each segment was one fifth of the stem height) and the diameter of each segment measured at the midpoint. The following measurements were taken in each primary, secondary, and high-order branch: length and 3 diameter measurements (on the bottom, middle and top). Height inaccessible diameters and branches were measured using a step ladder.

A basal wood section of no less than 10 cm in length was cut down from the thickest primary branch previously removed from each tree and its basic wood density determined. The volume of the stems and that of the branches were calculated using Hohenadl's and Newton formulae, respectively [3,5]. The biomass of each stem and branch was obtained by multiplying the volumes by the basic wood density of the basal wood section of the thickest branch.

The leaves of 3–5 primary branches (including the one removed from the tree for basic wood density determination) from all size classes were collected for dry mass determination. The biomass of the whole foliage was computed by multiplying the weighted mean dry mass of the leaves per branch (weighted by branch volume) by the number of primary branches in the tree. Crown biomass was obtained by summing the dry mass of each branch and that of the foliage. Aboveground biomass was obtained as the sum of crown and stem biomass.
